# Systematic evaluation of surgical insertion of flexible neural probe arrays into deeper brain targets using length modulation methods

**DOI:** 10.1088/1741-2552/ae385c

**Published:** 2026-02-02

**Authors:** Yingyi Gao, Zhouxiao Lu, Xuechun Wang, Zihan Jin, Alberto Esteban-Linares, Jeffery Guo, Huijing Xu, Kee Scholten, Dong Song, Ellis Meng

**Affiliations:** 1 Alfred E. Mann Department of Biomedical Engineering, University of Southern California, Los Angeles, CA, United States of America; 2 Ming Hsieh Department of Electrical and Computer Engineering, University of Southern California, Los Angeles, CA, United States of America; 3 College of Information and Electrical Engineering, China Agricultural University, Beijing, People’s Republic of China; 4 Elegen Corp, San Carlos, CA, United States of America; 5 Department of Mechanical Engineering, Purdue University, West Lafayette, IN, United States of America; 6 Bioventus, Valencia, CA, United States of America; 7 Department of Neurological Surgery, University of Southern California, Los Angeles, CA, United States of America

**Keywords:** flexible neural probes, flexible microelectrode array, neural probe insertion

## Abstract

*Objective*. Penetrating polymer-based microelectrode arrays (pMEAs) offer the potential for long term high-quality electrophysiological recordings of dynamic neural activity. Compared to rigid metal wire and silicon MEAs, improved device-tissue interface stability has been reported. However, accurate surgical placement of long, thin shanks in deeper brain regions is challenging as flexibility is achieved at the expense of axial stiffness. This study systematically evaluates then compares two pMEA placement strategies—dissolvable dip coating and molded brace, both with bare, exposed pMEA tips—to address the need for consistent, reliable, and accurate surgical targeting. These methods were selected based on the criteria of ease of fabrication, surgical feasibility, and mechanical performance. *Approach*. Sham (mechanical model with no electrodes) and fully functional pMEAs with shanks up to 5.5 mm long were fabricated and then modified using biodegradable polyethylene glycol (PEG) to support implantation. PEG was applied to shanks by motorized dip coating or a mechanical mold. Dissolution time and insertion in agarose gel brain models and rat cortex were evaluated followed by targeting of dip coated pMEAs to the rat hippocampus. *Main results*. Dip coating at high withdrawal speeds achieved uniform coating on shanks. Both strategies yielded similar critical buckling forces and insertion forces for single shank and arrayed pMEAs. Dip coated pMEAs were successfully placed in hippocampal regions without severe tissue damage as confirmed by histology and recordings obtained. *Significance*. Dip coating is a simpler method to prepare pMEAs for surgical targeting of deep brain regions compared to the bracing technique, as it does not require both a specialized mold and application process. This work provides a guide for researchers using single or multi-shank pMEAs to an accessible insertion strategy for implanting into deep brain regions in rodents and other small animal models.

## 1. Introduction

Advances in microfabrication and microelectromechanical systems technology have enabled miniature multielectrode neural interfaces for electrophysiological recordings with single unit resolution in different brain regions. As high quality long-term neural recordings lasting weeks to months to years are required to advance understanding of neural circuit function and dynamics, approaches that improve tissue-device interface stability are increasingly important. Polymer-based multielectrode arrays (pMEAs), in which the device is developed using a thin film polymer backbone, have been developed as an alternative to the prior generation of neural interface technology which utilized stiffer materials, such as silicon [1–6] and metals [7, 8]. The reduction in Young’s modulus of the polymer relative to that of brain tissue may mitigate tissue damage [9, 10] and the effects of chronic inflammation, including neuronal death and glial scarring [11–14].

Such neural interfaces share a similar format: a long, slender beam or array of beams, often referred to as probes or shanks, that each contain one or more electrode sites. Implantation relies on advancing the pMEA along a straight path from the initial insertion site to the targeted region with the aim of positioning electrode sites close to individual neurons. However, when the shank material is reduced in stiffness and as the beam length increases, the buckling resistance is also reduced. Therefore, the stiffer outer meningeal layers and nonhomogeneous composition of the brain may present a barrier to achieving implantation along straight trajectories to the desired targets [15, 16]. Microfabricated beams may exhibit curvature due to residual stress, presenting an additional challenge to accurate implantation [17]. Together, these factors have generally limited pMEAs to placement in shallow targets (<3 mm deep) [18].

Since there are many compelling deeper targets, such as thalamus and hippocampus, several methods have been proposed to accurately implant pMEAs to greater depths. These aim to enhance the maximum buckling force that a probe, modeled as a beam, can withstand during penetration into brain tissue, as represented by Euler’s beam equation:

\begin{align*}{F_{{\mathrm{buckling}}}} = \frac{{{\pi ^2}Ew{t^3}}}{{12{{\left( {kL} \right)}^2}}}\end{align*}

where *E* represents Young’s modulus, *w* and *t* are the width and thickness of the probe, respectively, *k* is the effective length factor, and *L* is the unsupported length of the probe.

The value of the effective length factor, *k*, depends on the probe boundary conditions and hence the relationship of the probe with the tissue [19–22]. Prior to insertion, the proximal end is held fixed (translation and rotation not possible) and the distal end is free (movable in translation and rotation) which sets *k* at 2. At initial contact of the probe tip but without tissue penetration, the distal end is hinged (translation not possible but rotation permitted) and *k* changes to 0.7. After tissue penetration, the distal end is fixed, and *k* is adjusted to 0.5.

If the insertion force exceeds buckling force, the probe is at risk of buckling and deflecting from intended targeting path. Conversely, if the insertion force is less, the probe can successfully penetrate the tissue. Variables *E, w, t,* and *L* can be adjusted to meet specific insertion requirements and improve resistance to buckling.

**Figure 1.**
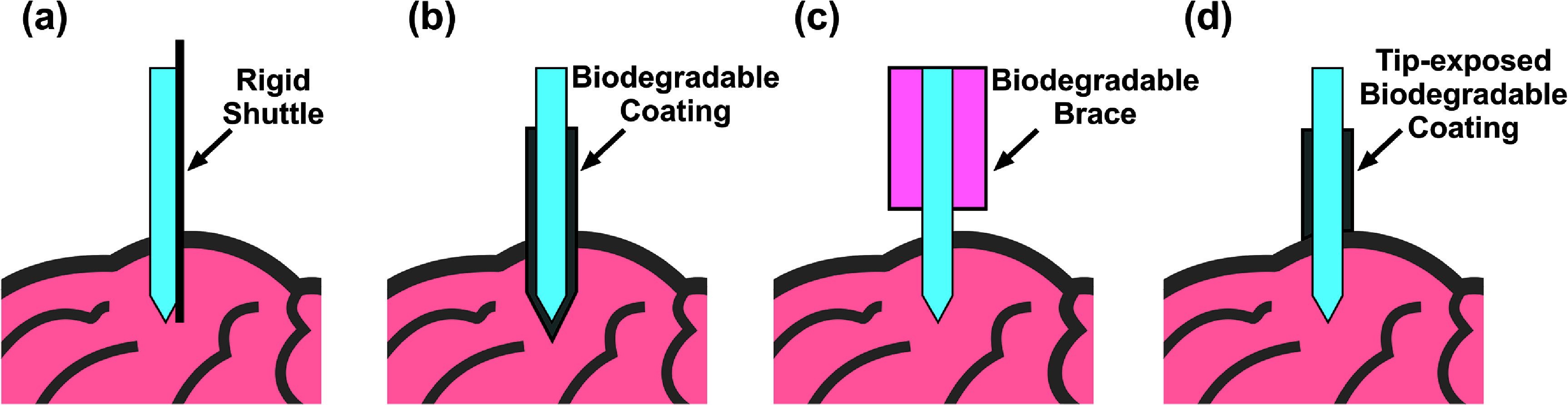
Illustrations depicting common approaches to achieve insertion of flexible pMEAS (light blue and viewed from side) into the brain: (a) rigid shuttle, (b) biodegradable coating, (c) biodegradable brace, and (d) tip-exposed biodegradable coating.

One common implantation strategy entails temporarily increasing the cross-sectional area (*w* and *t*) and stiffness (*E*) by attaching a rigid shuttle [23–26] or applying a dissolvable and biodegradable coating material as illustrated in figures 1(a) and (b). Although both methods produce an insertion track larger than the cross-sectional area of the probe alone, rigid shuttles require precise alignment and mounting followed by removal after probe placement, the latter risking additional damage and potential displacement of the probe from the targeted region during the retraction process [27–32]. Biodegradable coatings degrade over time, allowing the probes to regain their flexibility but at the cost of obscuring electrode sites. Coatings over a probe can be applied using a mold, which can be customized in dimensions and material [33–35]. Dip coating has also been explored; however, achieving uniform coatings and the desired thickness requires process tuning [19, 36–42].

Alternatively, a biodegradable brace can temporarily reduce the effective length (*L*) of a probe by exposing only a short length during the initial insertion and later exposing the entire length to complete its placement (figure 1(c)). This approach maintains the original probe cross-sectional area throughout implantation but requires a mold and additional processing steps to apply the brace. The brace must also be gradually dissolved as the probe is advanced to the target during the implantation process [18, 43].

Table 1 surveys and categorizes pMEA studies employing biodegradable coatings, braces, and rigid shuttles,which have largely focused on the insertion of single probes. However, multi-shank probes are essential for accessing larger brain volumes and more regions and only limited data is available on the insertion and mechanical characterization of closely spaced arrays [44, 45]. In addition, few studies have quantitatively compared different insertion methods; such comparisons are critically needed to arrive at best practices.

**Table 1.** Summary of studies employing biodegradable coatings,braces, rigid shuttles, or engineered cross section.

Insertion technique	Additional materials used	Native shank cross section (*µ*m^2^)	Supported shank cross section (*µ*m^2^)	Probe structural material	Shank number	Brain target and approximate depth (mm)	Durotomy	References
Molded coating	Silk	24 × 200	240 × 350	Parylene C	1	Mouse hippocampus (2–3.6 mm)	Yes	[33]
	Gelatin	125 × 250	130 × 400	Parylene C	1	Rat cortex (1–4 mm)	Yes	[34]
	PEG	20 × 90; 24 × 200	100 × 90; 236 × 350	Parylene C	4;1	Mouse cortex & hippocampus (1–3.6 mm)	Yes	[35; 33]

Dipped coating	Maltose	10 × 200	130 × 200	Polyimide	1	Rat hippocampus (2.45–5)	—	[19]
	Saccharose	14 × 160	164 × 218;	BCB; Parylene C	1	Rat cortex (1–4 mm)	Yes	[36];
	Dextran	14 × 500	163 × 641	Parylene C	1	Rat cortex (1–4 mm)	Yes	[37]
	PEG/PEG-PLGA mixture	20 × 180; 3 × 25; 3 × 170; 10 × 160; —	—	Parylene C	4;5;1;6;3	Mouse & rat, cortex & hippocampus (1–5 mm)	Yes	[38–42]

Dissolvable brace (via molds)	PEG	20 × 150; 10 × 90;	—	Parylene C	8;4	Mouse & rat cortex & hippocampus (1–5 mm)	Yes	[18; 43]

Rigid shuttles	Tungsten	15 × 120	+Ø 50 *µ*m	SU-8	1	Mouse hippocampus; dorsal striatum (1–3 mm)	No	[23; 24]
		30–100 *µ*m^2^	+Ø 25 *µ*m	SU-8	4–8	Mouse and Rat cortex and hippocampus (1–5 mm)	Yes	[25]
		20 × 350	+ Ø 50 *µ*m	Parylene C	1	Rat cortex (1–4 mm)	Yes	[32]
	Silicon	14 × 80	+30 × 80 *µ*m^2^	Polyimide	2	Rat OFC and NAc (4-7 mm)	No	[31]

Engineered Cross Section	Buckling-resistant and trace-stacked design	1225 *µ*m^2^	—	Polyimide	1	Rat cortex (1–4 mm)	Yes	[26]

PLGAPoly lactic-co-glycolic acid; PEGPolyethylene glycol; BCBBenzocyclobutene.

We previously reported on a dissolvable brace (figure 1(c)) and manual dip coating (figure 1(b)) to support implantation of multi-shank pMEAs [18, 38, 46, 47]. In this study, we developed a motorized dip coating method that greatly improved coating uniformity through control of withdrawal speed, is suitable for both single and arrayed pMEAs, and removes the coating at the tip (figure 1(d)). This enabled a head-to-head comparison of the motorized dip coating method and dissolvable brace method, evaluating material dissolution time and mechanical performance under *in vitro* and *in vivo* conditions. For both, polyethylene glycol (PEG) was selected as the stiffener material as it is widely available, is biocompatible, and offers tunable dissolution time. To simplify the fabrication process, sham devices mimicking the structure of pMEAs but lacking electrode features were used for non-recording experiments. Using our previously described prior rat hippocampal probe designed to record simultaneously from the CA1, CA3, and DG regions [18, 48], the motorized dip coating method was further evaluated in acute *in vivo* experiments.

## 2. Materials and methods

### 2.1. PMEA design

To systematically compare the two selected strategies, this study characterized the insertion of single and multi-shank (2, 4, and 8) sham pMEAs in benchtop brain models and *in vivo* in rat brain. Non-functional sham devices constructed only of Parylene C but otherwise having the same dimensions and layout of functional pMEAs were used for non-recording experiments. This simplification greatly reduced the time and expense to fabricate pMEAs while also providing similar mechanical properties; the metal layer containing the electrode sites and wiring is thin and does not change bulk pMEA mechanical properties (225 nm compared to 20 *µ*m).

The reference design, based on prior work [18, 48], used a sword-like probe shape terminating in a symmetric pointed tip with sides at a 45° angle. Individual shanks were 20 *µ*m thick, 5.5 mm long, and gradually transitioned from a wider base of 150 *µ*m width for a length of 4.125 mm, tapering to 110 *µ*m over 1.242 mm, and finally to a point within 133 *µ*m.

**Figure 2.**
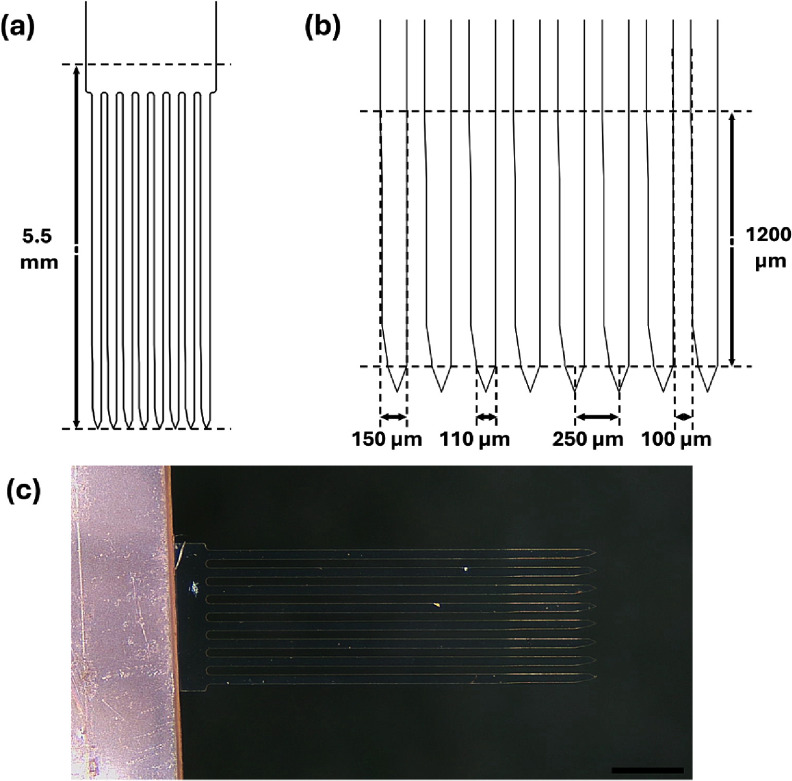
(a) Schematic showing overall layout of a 5.5 mm long, 8 shank array and (b) closeup view of tip region with dimensions of major features. (c) Photograph of a fabricated 8 shank Parylene sham array having 5.5 mm long shanks (scale bar = 1 mm).

This shape minimized device width across regions and resulted from using a single metal layer to contain electrodes and traces with safe margins between features. For linear arrays (2, 4 or 8 shanks), shanks were arranged side-by-side, with a 250 *µ*m center-to-center separation, and joined at the proximal end in a wider ribbon cable region for fanning out individual electrode traces for external connection (figure 2). The span of eight shanks was selected to fit within a 2000 *µ*m distance along the septal-temporal axis of the rat brain.

Similar devices were used for *in vivo* experiments (pMEA with four 5 mm-long shanks, 20 *µ*m in thickness, 180 *µ*m to 80 *µ*m in width, and center-to-center separation of 500 *µ*m) [47]. Each shank contained 16 electrodes, each 15 *µ*m in diameter, evenly distributed along both edges in a staggered arrangement for a total of 64 electrodes in the 4-shank array. Along the shank axis, the center-to-center distance between the electrodes was 120 *µ*m. The recording region spanned 1800 *µ*m along the shank axis to reach CA1, CA3, and DG regions of the rat hippocampus. Each Pt electrode was connected to a 3 *µ*m wide trace running along the shank to wider traces terminating in an external contact pad. The 8 × 8 mm^2^ proximal end contained 64 regularly spaced contact pads supporting direct bonding to a matching 18 × 17 mm^2^ printed circuit board (PCB) using the polymer ultrasonic on bump (PUB) bonding method [49].

### 2.2. PMEA fabrication

The shams were fabricated using microlithographic techniques on a bare 100 mm (4”) diameter silicon carrier wafer. Wafers were pre-baked at 110 °C for at least 10 min to dehydrate the surface. Two 10 *µ*m thick layers of Parylene C (Specialty Coating Systems, Indianapolis, IN) were sequentially deposited (PDS 2010 Labcoater, specialty coating systems, Indianapolis, IN) to obtain a 20 *µ*m thick film, mimicking the dual deposition required to produce the fully functional pMEAs. AZ 4620 photoresist (AZ Electronic Materials, Branchburg, NJ) was spin-coated in two layers (5 s @ 500 rpm; 45 s @ 1000 rpm), forming a 30 *µ*m thick etch mask outlining the shams. Parylene C was then etched using a switched chemistry process of C_4_F_8_/O_2_ in a deep reactive ion etcher (Oxford Plasma Lab System 100; ∼230 loops, 700 W ICP, 20 W RF power, 23 mTorr [50]). The photoresist was stripped in sequential acetone, isopropyl alcohol, and deionized (DI) water rinses. Devices were released by soaking in DI water and gently peeling away from the native oxide layer on the silicon substrate. The released shams were annealed to enhance the entanglement of the Parylene C chains and alleviate internal stress [51]. Annealing was performed with shams sandwiched between two ceramic plates in a chamber vacuum purged three times with nitrogen (N_2_) then held at 200 °C for 48 h [52].

Functional pMEAs were micromachined on a bare 100 mm (4”) diameter Si wafer in a layer-by-layer process [47]. The detailed process protocol is available at [47, 53]. Briefly, a 10 *µ*m Parylene C was deposited, followed by a Ti/Pt/Au/Pt stack of 20/25/155/25 nm. This metal layer formed the electrode sites, connecting traces, and contact pads and was defined by lift-off. A second layer of 10 *µ*m thick Parylene was deposited to encapsulate the metal layer. Openings for the electrode sites and contact pads were created using the same switched chemistry process as for the shams and then repeated to define the pMEA outline. The devices were then released and annealed using the same process as for the shams. The integrity of electrodes and traces was inspected under a microscope and the electrochemical properties of individual electrodes were verified using electrical impedance spectroscopy to qualify devices for *in vivo* experiments. Devices were attached to a PCB using PUB bonding. Two 36-pin connectors (Omnetics A79024-001, Minneapolis, MN) soldered to either side of the PCB provided external connections to a data acquisition system.

### 2.3. PMEA preparation for mechanical evaluation and surgical insertion

#### 2.3.1 Dissolvable brace

To consistently define the brace on each sham, a three-layer polydimethylsiloxane (PDMS, Sylgard 184, DOW Corning Corp., Midland, MI) mold fabricated using a vinyl cutter (Graphtec® cutting plotter CE6000-40, Irvine, CA) with a 0.5 mm thick acrylic backing was used. Each PDMS layer and the resulting brace were 0.5 mm thick. Total brace volume was determined from the mold dimensions.

**Figure 3.**
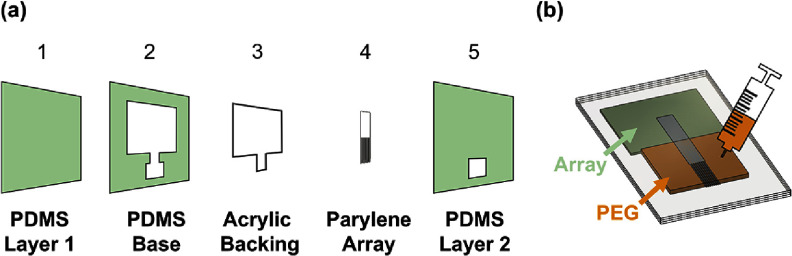
Illustration depicting the (a) layers used to construct the mold and (b) process to apply a brace to the mold-defined region. This example shows the process for an array; the same process can be used for a single probe.

After placing the sham into the mold with 1–2.8 mm of the tip protected, the assembly was warmed at 60 °C for 3–5 min. PEG (3350 MW) was melted at 60 °C and poured into the mold opening. A PDMS cover was then applied to obtain a uniform thickness across the brace (figure 3). The assembly was cooled down to room temperature, allowing the PEG to solidify, and the braced pMEA released by gently peeling away the mold from the proximal to the distal end.

PEG 3350 MW was selected to balance the dissolution time (at a rate of approximately 5.5% per minute) and integrity of the brace against premature softening due to water absorption during implantation [19].

#### 2.3.2 Dip coating

To ensure repeatable coating thickness, shams were withdrawn at controlled speeds on a motorized linear stage from the coating bath [38, 42]. The interplay between entraining and draining forces governs the final film thickness, which can be theoretically explained by the Landau–Levich theory [54, 55].

We use the theory for a fiber, modified to our case in which a shank resembles a beam, to represent the final film thickness *h*

\begin{equation*}h = 1.34\frac{w}{2}{\left( {\frac{{\eta U}}{\gamma }} \right)^{{\raise0.7ex\hbox{$2$} \!\mathord{\left/ {\vphantom {2 3}}\right.} \!\lower0.7ex\hbox{$3$}}}}\end{equation*}

where *w* is the width of the beam, *η* the solution’s dynamic viscosity, *U* the withdrawal speed, and *γ* the surface tension. This relation shows that thickness scales with withdrawal speed as

\begin{equation*}h \propto \,{U^{{\raise0.7ex\hbox{$2$} \!\mathord{\left/ {\vphantom {2 3}}\right.} \!\lower0.7ex\hbox{$3$}}}}.\end{equation*}

Therefore, thickness grows in a sublinear pattern with withdrawal speed. At very low speeds, however, evaporation can dominate over viscous entrainment, leading to non-uniform coatings. To obtain consistent coatings, sufficiently high withdrawal speeds should be maintained. Further discussion about this process and related phenomena are discussed in §4.

**Figure 4.**
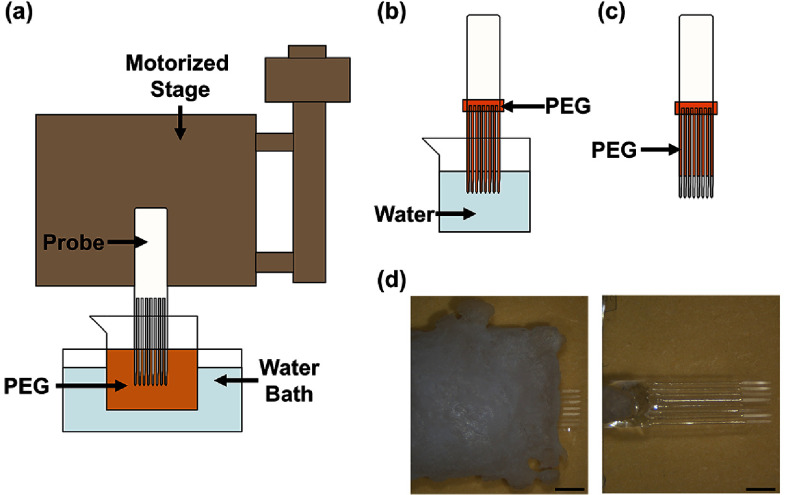
(a)–(c) Illustration of the motorized dip coating setup and process steps. A sham array is mounted to the motorized stage and lowered into a container of molten PEG housed in a temperature regulated water bath. The PEG coated sham array is then immersed in water to dissolve the coating at the tip then retracted. (d) Photographs showing braced and dip coated probes with the tip exposed (scale bar = 1 mm).

Shams were vertically aligned, affixed to the motorized stage (Thorlabs LNR502, Newton, NJ), and the distal tips lowered into a molten PEG solution (3350 MW, maintained in 70 °C–80 °C water bath) (figure 4(a)). Either 4.5 mm length measured from the tip, or the entire length were exposed to PEG solution. After soaking for 10–30 s to ensure consistent coating contact across the sham surface, withdrawal was initiated at a constant speed (0.1–2.5 mm s^−1^). Coatings solidified at room temperature for 10–20 min. To further enhance the stiffness of the probe, PEG 8000 MW was applied to the proximal end manually for approximately 1 mm. Then a 1 mm or 2.8 mm length from the tip was exposed by submerging to that depth in DI water for 10 s and retracting with the assistance of the motorized stage (figures 4(b) and (c)). Each coated probe was subsequently examined under a microscope to determine the thickness and verify uniformity and coverage. The thickness of the PEG coating was quantitatively measured using NIH ImageJ software (version 1.54d) and used to calculate its volume by multiplying with length and width.

#### 2.3.3 Evaluation of dissolution time

The dissolution time for the PEG brace and coating was determined for the 8 shank shams. For each case, the first 1 mm from the shank tip was exposed (*n* = 3). To facilitate visualization, PEG was mixed with blue food dye. Dissolution was observed during immersion in 3 ml of 1× phosphate buffered saline which mimics the intracortical environment. The dissolution with only the first 4.5 mm as measured from the tip and the full length was recorded until the PEG was completely dissolved. To examine the impact of the coating and dissolution process on the device’s surface energy, contact angle of a droplet of DI water applied to the sham was measured (*n* = 3) before and after PEG was applied.

### 2.4. PMEA experimental evaluation methods

#### 2.4.1 Agarose brain phantom preparation

Insertion studies were performed in 0.6% agarose hydrogel (Sigma-Aldrich, Burlington, MA) blocks [56] which were selected as the mechanical properties closely mimic that of brain tissue [57]. Gel was prepared by dissolving 0.72 g of agarose in 120 ml of DI water in a laboratory microwave at power level 10 for 1 min, followed by additional heating in 10–30 s intervals until the solution became transparent [58].

To form consistent blocks suitable for optical viewing, the molten agarose was poured into a transparent plastic box and the surface covered with a glass slide to avoid meniscus formation and obtain a flat surface upon solidification. After approximately 10 min of cooling and solidification at room temperature, the mold was sealed with parafilm to prevent contamination and dehydration and stored in a laboratory refrigerator. Prior to use, the weight of the agarose gel was measured to ensure consistent agarose composition. Any gel showing a weight loss exceeding 10% was discarded.

#### 2.4.2 PMEA mechanical evaluation with load cell and in agarose

Insertion and buckling forces were determined using 8 shank shams with shanks selectively removed to obtain devices with fewer shanks (1, 2, 4, 8; *n* = 4). Since insertion force is independent of probe length, shorter probes were utilized to facilitate experiments. These test structures were prepared by covering all but a 2.8 mm length of the tip, using motorized dip coating followed by selective dissolution (as described in §2.3.2).

**Figure 5.**
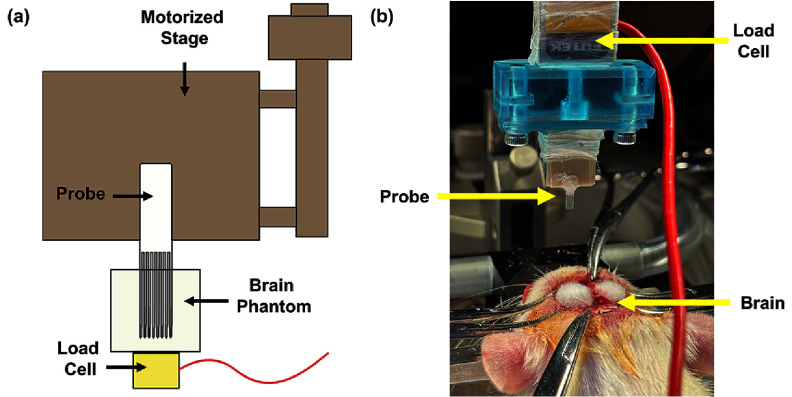
(a) Schematic and (b) photograph showing the mechanical testing setup for force measurement during insertion into the agarose model and rat brain, respectively.

After mounting on a motorized linear stage, shams were driven at a constant speed of 0.01 mm s^−1^ into an agarose block positioned on a calibrated 50 g load cell (Futek FSH03869, Irvine, CA) (figure 5(a)). This speed was chosen to minimize shear forces on the surrounding brain tissue, as supported by previous studies [14, 18, 59]. Force data was continuously collected, and the insertion force was determined by the initial peak force required to pierce the surface.

To determine buckling force, shams were mounted to the load cell and driven against a metal plate at a constant speed of 0.01 mm s^−1^ [60–64]. The force exerted was continuously recorded as a function of stage displacement and the buckling force designated as the force at which all shanks of the array buckled.

#### 2.4.3 PMEA evaluation in vivo

To complement the benchtop experiments, insertion force was also measured *in vivo* using single and 8-shank shams prepared with the brace or dip coating. In all cases, a 1 mm length measured from the shank tip was left exposed and uncoated.

All animal experiments were reviewed and approved by both the Institutional Animal Care and Use Committee and the Department of Animal Resources of the University of Southern California. The surgical procedure [48] used 10–12 week old Sprague–Dawley rats anesthetized with 3%–4% isoflurane for induction and 1.5–2.5% isoflurane for maintenance during the surgery. A stereotactic frame with ear bars was used to secure the animals. The cerebral cortex was exposed using a 2.5 × 4 mm^2^ window (1.5–5.5 mm caudal to the bregma and 1.5–4 mm lateral of the sagittal suture). Both dura and pia mater were then carefully removed with forceps. Five screws were anchored into the rat skull around the surgical window. The exposed brain was protected with a cotton ball soaked in saline prior to pMEA implantation.

To measure insertion force during implantation, a custom 3D printed resin block (securely mounted on the stereotaxic arm with Parafilm) was used to attach the pMEAs and the 50 g load cell (figure 5(b)). The probes were advanced at a speed of 0.01 mm s^−1^ down to a depth of 1–2 mm (measured from probe tip contact with the brain surface) while monitoring insertion with a microscope.

Functional Parylene pMEAs (*n* = 11) were also prepared using the motorized dip coating method and implanted using the same surgical protocol. The pMEAs were vertically aligned and secured to the stereotaxic frame in the PCB region using Parafilm. Shanks were advanced into the brain to a depth of 4–5 mm from the surface at a speed of 0.01 mm s^−1^. Saline was applied to dissolve the PEG added at the proximal end when the probe was inserted deeper than 4 mm.

During insertion, electrode sites were continuously monitored for the presence of complex spikes [38] to confirm placement in the CA1, CA3, and DG layers of the hippocampus. Once the pMEA reached the targeted regions, dental cement was applied at the insertion site up to the PCB to secure the device. At one-month post-implantation, the animal was euthanized and perfused with paraformaldehyde. The rat brain was dissected from the cranium, the pMEA was removed, and the tissue was dehydrated overnight in 18% sucrose. The dehydrated tissue was then embedded in optimal cutting temperature compound, mounted onto a cryostat set at −20 °C, sectioned into 50 *µ*m thick slices, and prepared for histological staining.

To quantitatively compare tissue responses between the two insertion approaches, immunohistological analyses were performed on sham-implanted arrays: 8-shank arrays inserted using the brace method (staining protocol described previously [48]) and 4-shank arrays implanted using the dip-coating method. On post-implantation day 4, the animal was euthanized and transcardially perfused with 50 mL of cold phosphate buffered saline (PBS, pH 7.4) at 5 ml min^−1^, followed by 50 ml of 4% formaldehyde (FA) in PBS at the same rate. The right hemisphere was then dissected, the array was removed, and the tissue was post-fixed in 4% FA in PBS for 24 h at 4 °C.

Tissue dehydration, clearing, and paraffin infiltration were performed with the following sequence: 70% ethanol (1 h), 80% ethanol (1 h), 96% ethanol (3 × 1 h), 100% ethanol (2 × 1 h), xylene (2 × 1 h), and paraffin (4 × 1 h). The paraffin-infiltrated tissue was embedded, cooled, and sectioned on a microtome into 15 *µ*m slices. Sections corresponding to ∼2.7 mm from the superior brain surface were subjected to double immunohistochemistry using antibodies against glial fibrillary acidic protein (GFAP; Red) and neuronal nuclei (NeuN; DAB (3,3’-diaminobenzidine)) to visualize astrocytes and neurons, respectively.

For quantitative analysis, control regions were selected from the same histological sections. Concentric radial rings (25 *µ*m width) were drawn around the array cross-sections and corresponding control regions. Astrocyte density within each annular ring was quantified using ImageJ.

## 3. Experimental results

### 3.1. Motorized dip coating characterization

**Figure 6.**
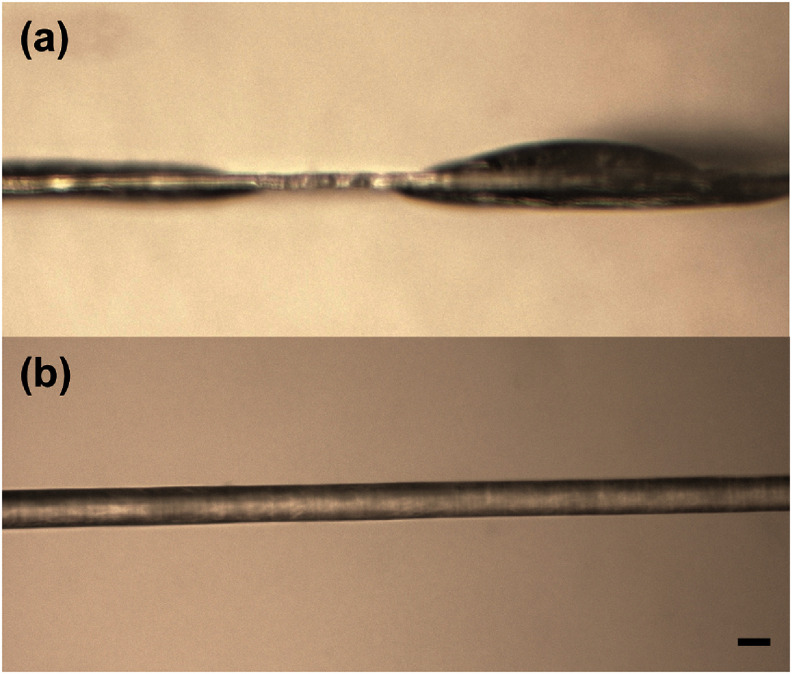
Representative closeup photos showing side views of (a) a nonuniform coating produced at 0.5 mm s^−1^ withdrawal speed and (b) a uniform coating produced at 1.5 mm s^−1^ withdrawal speed on single Parylene shanks (scale bar = 60 *µ*m).

**Figure 7.**
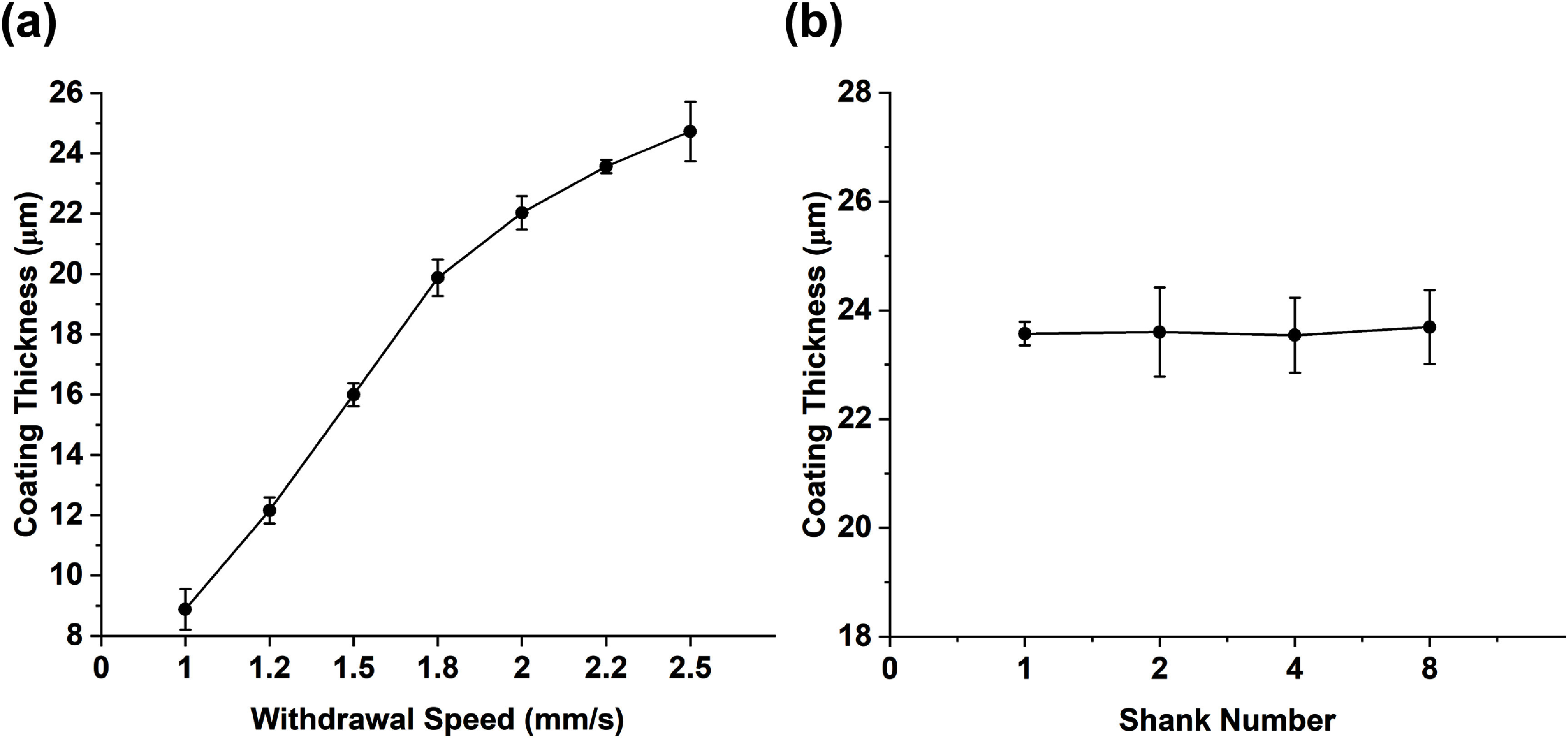
(a) PEG coating thicknesses as a function of withdrawal speed between 1 to 2.5 mm s^−1^ (mean ± SD, *n* = 3). (b) PEG coating thicknesses at 2.2 mm s^−1^ withdrawal on shams with different numbers of shanks (mean ± SD, *n* = 3 for 1 shank, *n* = 6 for 2 shanks, *n* = 12 for 4 shanks, *n* = 24 for 8 shanks (where n refers so the number of shanks and 3 shams were used for each configuration)).

Nonuniform PEG coating along the length of shanks was observed at withdrawal speeds less than 1 mm s^−1^. At speeds of 0.1 and 0.5 mm s^−1^, discontinuous clumps were observed at irregular intervals along the shank length (figure 6(a)). At speeds ranging from 1 to 2.5 mm s^−1^, uniform coating along the shank length (figure 6(b)) was achieved, with the average coating thickness, as measured on one side of the probe, increasing three-fold from 8.8 ± 0.68 to 24.73 ± 0.98 *µ*m, as shown in figure 7(a).

For 4- and 8-shank arrays with the entire length dip coated, adhesion between neighboring shanks was observed, whereas adhesion was not observed when only the first 4.5 mm was coated. Arrays withdrawn at a rate of 1.5 mm s^−1^ and above (approximately 16 *µ*m thick coating) with the first 2.8 mm of the tips exposed were successfully inserted into agarose. However, the insertion of arrays coated using lower withdrawal rates into agarose was not consistently achieved. For insertion into rat brain, successful insertion was achieved with arrays withdrawn at a rate of 2.2 mm s^−1^ and above (approximately 23.5 *µ*m thick coating). At this rate, the coating thickness was independent of the number of shanks in the array (figure 7(b)).

### 3.2. Evaluation of dissolution time

The coating volume resulting from each coating condition was calculated and dissolution time for each measured using 8 shank shams (table 2). Since the proximal end of the dip coated probe was manually reinforced with PEG 8000 MW, the coating dimensions at this portion of the sham varied.

**Table 2.** Comparison of coating volume and dissolution time between coating methods.

Method	Coating volume (mm^3^)		Dissolution time (minutes)	
	3.5 mm length	Entire coating	3.5 mm length	Entire coating
Brace	22.32	28.69	18.86 ± 2.07	21.78 ± 2.17
Motorized dip coating	0.31	0.31 + V^a^	0.27 ± 0.02	6.19 ± 1.88

^a^ *V* = volume of manually coated PEG.

The contact angle before applying PEG was 82 ± 2.30°. After the PEG dissolved, the angles for each method were measured as 81.33 ± 1.15° and 80.67 ± 1.15°, indicating minimal change in surface energy after PEG was fully dissolved.

### 3.3. PMEA mechanical evaluation with load cell and in agarose

**Figure 8.**
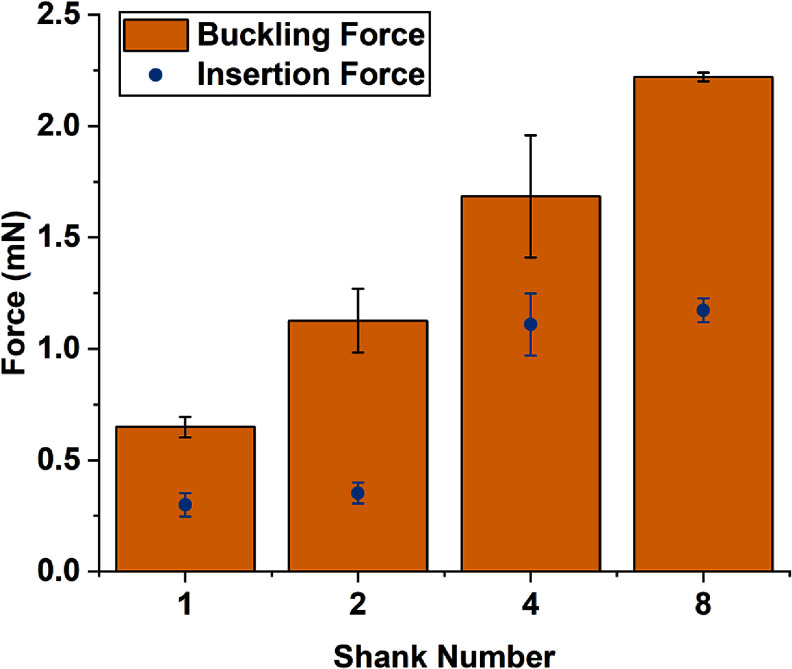
The measured buckling (mean ± SD, *n* = 4) and insertion forces (mean ± SD, *n* = 4) for single shank probes and linear shank arrays with 2.8 mm of tip length exposed.

Figure 8 shows the buckling (increased from 0.65 ± 0.05 to 2.22 ± 0.02 mN) and insertion (increased from 0.30 ± 0.05 to 1.17 ± 0.12 mN) forces recorded for coated probe arrays with up to 8 shanks shortened with PEG. These forces were comparable to those previously reported for PEG braced probes inserted into agarose [22]. This correlation is supported by Euler’s equation, given that the exposed length was consistently 2.8 mm for both methods. For larger arrays, the buckling forces were close to the insertion forces, suggesting that the arrays should be further shortened during *in vivo* implantation to prevent buckling.

### 3.4. PMEA evaluation *in vivo*

#### 3.4.1. PMEA mechanical evaluation with load cell and in vivo

The nonparametric Mann–Whitney U test was used to compare the different groups, considering the small sample size [59]. Variances between groups were evaluated using Levene’s test with significance determined by a *p*-value less than 0.05.

**Figure 9.**
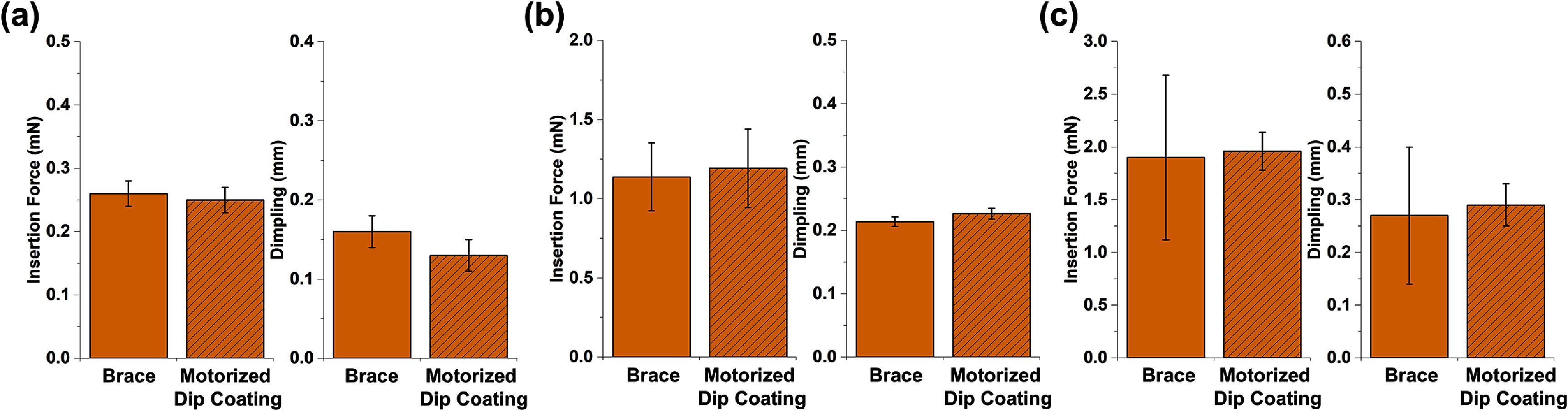
The insertion force of PEG braced and motorized dip coated shams in rat brain measuring 5.5 mm long and with 1–2 mm of the tip length exposed for (a) single probes (mean ± SD, *n* = 5 for both methods), (b) 4-shank arrays (mean ± SD, *n* = 5 for brace, *n* = 7 for dip coating) and (c) 8-shank arrays (mean ± SD, *n* = 5 for both methods).

Figure 9 shows the insertion forces recorded and depth of dimpling observed for the dip coating and brace methods in both single, 4-and 8-shank probes. For the dip coating method, the insertion force increased from 0.25 ± 0.02 to 1.96 ± 0.18 mN from single to 8-shank probes. For the brace method, the insertion force increased from 0.26 ± 0.02 to 1.90 ± 0.78 mN. A comparison between the two methods identified no statistically significant differences (*p* > 0.05).

#### 3.4.2. In vivo demonstration of motorized dip coating method in rat hippocampus

**Figure 10.**
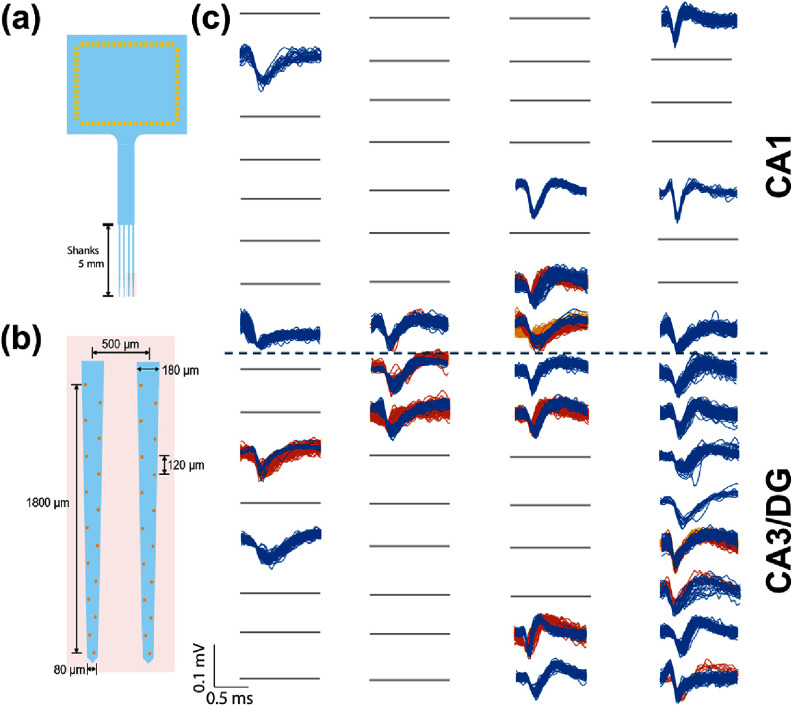
Acute multi-region recordings from a functional pMEA implanted using the dip coating technique. (a) Schematic of overall pMEA layout having 5 mm long shanks and (b) inset showing magnified view of electrode arrangement near the probe tip. Major feature dimensions are indicated. (c) Representative waveforms of unitary activities.

**Figure 11.**
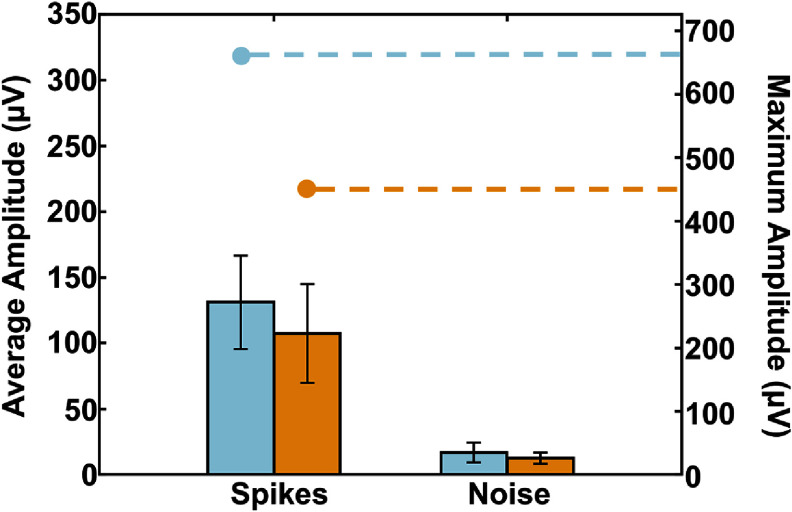
Average complex spike amplitudes and noises levels across all three braced array implantations (light blue bars) compared to those from three dip coated array implantations (orange bars) mean ± SD, *n* = 3 for brace, *n* = 3 for dip coating). The maximum spike amplitude recorded for brace and dip coated array implantations is noted by the circle above each bar with connects to the *y* axis on the right with dashed lines.

**Figure 12.**
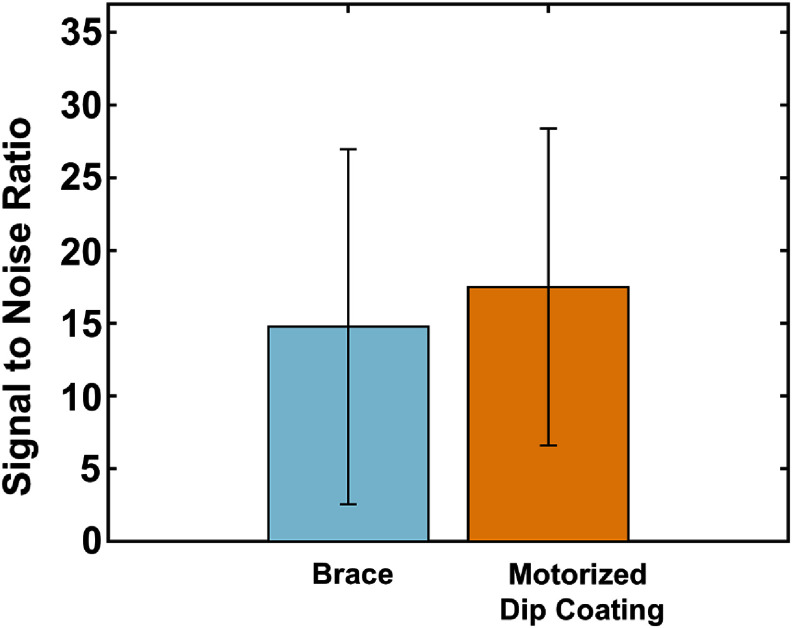
Average signal to noise achieved across all three array implantations (mean ± SD, *n* = 3 for brace, *n* = 3 for dip coating).

**Figure 13.**
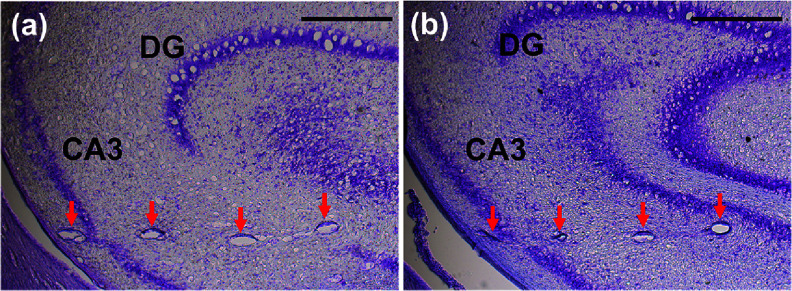
Histological slices in the hippocampal region after pMEA removal. Slices were stained with hematoxylin and eosin. Transverse slices taken at depths of (a) 3.46 and (b) 3.86 mm from the brain surface capture the presence of tracks left by the implanted pMEA shanks (red arrow). Scale bar is at 500 *µ*m.

The total duration of the surgical implantation process was approximately one hour after alignment to the target site and included time to acquire electrophysiological recording of complex spikes which provided confirmation of placement in the hippocampus. Representative spike waveforms from multiple units recorded from one animal are shown in figure 10. The hippocampal complex spikes amplitude, noise level, and signal-to-noise ratio (SNR) for the brace and dip coating methods are summarized in figures 11 and 12. A direct comparison of SNR between the two methods showed no statistically significant difference (*p* > 0.05). All four shanks obtained simultaneous multi-region recordings from the CA1, CA3, and DG. Histological brain sections (figure 13) demonstrated that the motorized dip coating method effectively facilitated insertion of a multi-shank pMEA to the targeted regions of the hippocampus.

**Figure 14.**
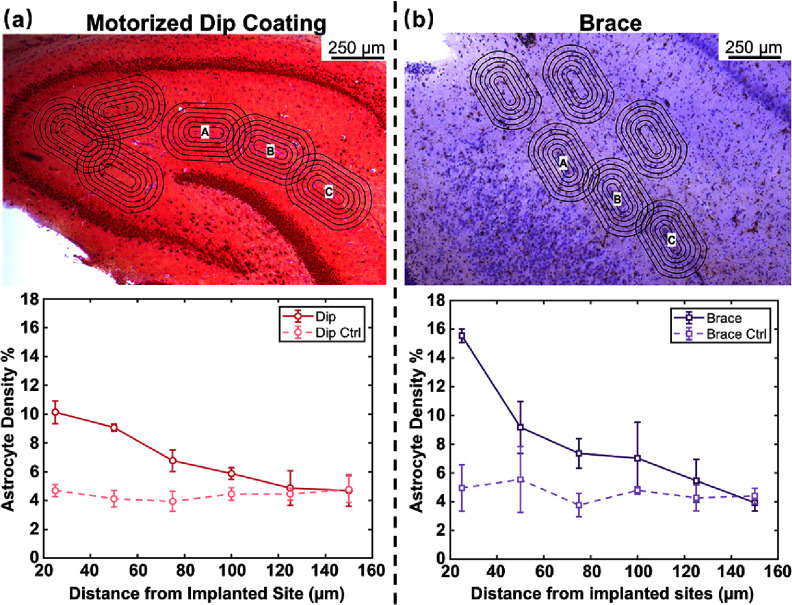
GFAP-labeled astrocyte distributions and quantitative density gradients for dip coated vs. braced implants. (a) Upper row: transverse slice implanted with a motorized dip coated sham array, double stained for NeuN (brown) and GFAP (red) at 2.7 mm depth with 25 *µ*m concentric rings around central implant sites A, B, and C (scale bar = 250 *µ*m). Bottom row: Astrocyte density as a function of distance from dip coated implant sites and corresponding control regions. (b) Upper row: transverse slice implanted with a braced sham array, stained for GFAP (brown) with hematoxylin (purple) counterstain at 2.7 mm depth with 25 *µ*m concentric rings around central implant sites A, B, and C (scale bar = 250 *µ*m). Bottom row: Astrocyte density as a function of distance from braced implant sites and corresponding control regions.

Figures 14(a) and (b), upper rows, show representative histological images from implanted and control regions, with astrocytes labeled in red using GFAP and neurons labeled in brown using NeuN. Astrocytes typically proliferate or hypertrophy in response to mechanical injury, and increased astrocytic labeling around the implant site is therefore a common indicator of glial reactivity to the implanted array. In both insertion conditions, a modest elevation in astrocyte density was observed relative to adjacent non-implanted tissue. Quantification of astrocyte density within concentric radial rings surrounding each probe track, along with corresponding measurements from matched control regions, is shown in the bottom rows of figures 14(a) and (b). For both insertion methods, astrocyte density decreases with increasing distance from the center of the implant site and approaches control levels within 150 *µ*m from the track boundary. Because the tissue sections collected from the brace-implanted hemisphere were slightly thicker (35 *µ*m) than those from the dip-coated hemisphere, the absolute number of labeled cells appears marginally greater in the brace group; however, relative spatial trends remain comparable. Additional immunohistological replicates are planned to more comprehensively assess the tissue response associated with each insertion technique over time in chronic studies. These initial results provide a useful baseline for future evaluations of long-term biocompatibility in length-modulated implantation strategies.

## 4. Discussion

This study describes a controlled process by which single and multi-shank pMEAs can be temporarily stiffened using a biodegradable polymer to achieve surgical implantation to the targeted brain region. Here, the hippocampus was selected as a deep structure for demonstrating the method. Expanding on prior studies using a molded brace and manual dip coating, a motorized dip coating process was developed to achieve uniform and reproducible PEG layers through precise control of withdrawal speed. Unlike manual coating, which often produces weak, nonuniform regions prone to buckling, the motorized method ensures consistent thickness, and therefore mechanical support, along the entire shank. In addition, the PEG coating at the shank tips was selectively removed, in part adopting the same principle used in PEG braces, in which only the bare probes are inserted in the brain and the coating is successively dissolved along the length during advancement. These refinements highlight the importance of PEG coating uniformity in achieving successful implantation and establish motorized dip coating as a simple, reproducible strategy for precise, reliable implantation of pMEAs targeting deeper brain structures in rodents.

### 4.1. Effect of withdrawal speed on the coating quality

When performing liquid dip coating with the goal of depositing a uniform film, the withdrawal parameters are critical. The deposited thickness and film quality depend on the interplay between withdrawal and drying; film formation is subject to entrainment and draining forces in addition to drying. There are distinct regimes defined by the withdrawal speed of the object to be coated: capillary (low speed), intermediate, and draining (high speed). In the draining regime, high withdrawal speeds are generally >1 mm s^−1^ and drying dominates over evaporation. Uniform, constant thickness coatings can be obtained with thicker films achieved at higher withdrawal rates. In the capillary regime, withdrawal speeds are generally <0.1 mm s^−1^. Evaporation rate dominates over drying; as the film solidifies on the surface, more material is driven onto the surface by capillary feeding. While thicker films are possible, defects such as the ‘coffee-ring effect’ may be present, preventing uniform coatings. In this scenario, the coating tends to pin at the edge of the meniscus between the deposited film and liquid, forming periodic thicker regions [54, 65].

In preliminary PEG dip coating experiments (3350 MW) on sham pMEAs, withdrawal speeds below 1 mm s^−1^ produced coatings that exhibited capillary regime behavior, whereas uniform coatings were observed for speeds above than 1 mm s^−1^, with withdrawal speed dependency as expected for the draining regime. For withdrawal speeds between 1 to 2.5 mm s^−1^, thickness of the PEG film increased, aligning with the thicknesses used previously in molded braces [34, 66]. It is important to note that factors such as solution viscosity, environmental conditions (temperature, airflow, cleanliness), and probe roughness influence coating thickness and quality and should therefore be controlled to ensure repeatability. Contact angle measurements may provide a starting reference to calibrate a coating process across different pMEA batches.

PEG is available in an assortment of higher and lower molecular weights, allowing finer tuning of dissolution time and therefore implantation time [18]. Thus, a user can simply select an alternative molecular weight (polymer chain length) to fine tune the implantation process based on their specific needs. Specifically, for the dip-coating method, 3350 MW PEG was applied to the shanks, whereas 8000 MW PEG was used at the proximal end of the pMEAs to provide additional mechanical rigidity. Withdrawal speed and coating thickness require further calibration if selecting a different PEG formulation for dip coating or if using another coating material with a viscosity different from that reported here. For the brace method, 3350 MW PEG was selected because lower molecular weights such as 1000–2000 MW dissolved too quickly for reliable handling, whereas PEG with molecular weight ⩾ 8000 dissolved too slowly for practical implantation times.

This study was limited to investigating pMEAs having different numbers of shanks while holding the shank geometry and shank-to-shank spacing constant. For the non-functional devices used in benchtop and *in vivo* mechanical tests, we used a 250 *µ*m center-to-center spacing to match our prior braced arrays and enable direct comparison of insertion performance. In contrast, the functional hippocampal pMEAs had four shanks with 500 *µ*m spacing and a different electrode layout tailored to the CA1, CA3, and DG targets. Across gel phantom and *in vivo* experiments, we did not observe noticeable differences in insertion behavior (for example, depth reached without buckling, dimpling, or deformation) between the 250 *µ*m and 500 *µ*m configurations, suggesting that within this spacing range, the mechanics are dominated by shank stiffness and coating rather than lateral spacing. For arrays with the same shank spacing but shorter shank length, or with the same length but larger shank spacing, less fusing between neighboring shanks is expected during dip coating. While pMEAs having more closely spaced shanks are possible, narrower spacing may damage cell bodies and reduce recording quality. Additionally, surface tension forces present during the dip coating process may cause increased adhesion of neighboring shanks and require additional calibration of the coating process.

### 4.2. Comparison between dissolvable brace and dip coating method

To enable direct comparison with the molded brace method, we held the probe geometry constant in the benchtop studies. A notable difference between the methods is that the brace required a greater volume of PEG and, consequently, more time was required for complete dissolution. Therefore the increased volume allowed greater flexibility during implantation surgery by extending the time that the pMEA retained added stiffness. However, additional surgical time was also required for sequential PEG dissolution by saline rinse, extending cranial window exposure time. In contrast, the dip coating method significantly reduced the coating volume and dissolved immediately upon contact with the hydrated brain surface.

Factors such as tip angle, shank thickness, and material coating have been reported to influence insertion dynamics, including insertion force [54, 59, 67]. Here, minimal differences in mechanical performance were observed when comparing across brace and dip coating methods for the same pMEA design. While previous *in vivo* studies on insertion dynamics mostly reported results for single shank probes made of metal or silicon [62, 68, 69], this report systematically evaluated single shank polymer probes and arrays in the same study, providing a baseline for future studies that may investigate other parameters such as shank length or three dimensional arrays.

Insertion force measurements of arrays exhibited greater variance than those of single shanks, which may reflect slight differences in tip contact across the length of an array attributed to the curvature of the cortical surface and heterogeneity in the composition of the brain, and therefore the mechanical properties, along any particular shank path. However, when implanting in rat hippocampus, this variance did not impact the successful insertion of arrays. The recorded spike amplitudes and histological results were comparable to those reported in the previous study using the dip coating method to implant functional pMEAs [38].

For successful insertion using either braces or dip coating, completely flat shanks are required, as curved shanks may deviate from the surgical path during insertion. Therefore, mechanical stress in fabricated pMEAs must be carefully controlled.

For the studies reported here, the meningeal layers had to be removed before surgical implantation to prevent probe buckling, as the absence of pia layers significantly reduced the penetration force required [70–73]. However, while this practice is common (table 1), the resulting meningeal inflammation, tissue death, and ejection of implanted devices, may adversely affect the chronic recording stability [74]. Additional reinforcement is required to prevent buckling while leaving the meningeal layers intact and requires further investigation.

Overall, the goal of reinforcing softer pMEAs is to achieve accurate placement while minimizing collateral tissue damage. The strategies reported and compared here focus on providing additional stiffness to shanks to overcome mechanical resistance during the implantation procedure while maintaining their native cross-section once within the parenchyma by ensuring that the PEG dissolves upon insertion. Such an approach may reduce early micromotion-induced stress, which is a factor in attaining chronic interface stability, compared to approaches that use coatings that remain on the shanks and dissolve within the tissue over time [75, 76].

In addition to PEG, several other materials, including maltose, gelatin, and silk, and reinforcement process have been explored (table 1). The specific choice should be tailored to pMEA design and study intent. PEG is a convenient choice as it is widely available in a broad range of molecular weights which allows tuning of dissolution time (from seconds to minutes) and stiffness. Maltose and saccharose are generally available in a single molecular-weight form and dissolve rapidly upon contact with cerebrospinal fluid, limiting their ability for stepwise modulation of insertion mechanics, while dextran and silk can persist longer from minutes to depending on molecular weight and formation, but may require more complex processing [19, 77].

More rigid shuttles such as tungsten wire and silicon shanks can penetrate the tougher meningeal layers. These shuttle-based approaches have been used by several groups for polymer probe implantation in non-human primates, where the intact dura and pia present a substantially higher mechanical barrier than in rodents and where deeper targets typically require probe lengths exceeding 5 mm [78–80]. These techniques provide sufficient stiffness to traverse the meningeal layers and reach deep structures but introduce added constraints. Wires need to be individually applied if multiple shanks are used and may even require customization of both the wire and pMEA for secure attachment [81, 82]. Silicon shuttles require a separate fabrication process and are not widely available. Rigid shuttles need to be inserted along with the pMEA and therefore increase the cross-sectional dimensions of each shank. In rodent models, the motorized dip-coating approach provides an alternative method that can be applied in a simple process to one or more shanks and even multiple devices simultaneously, while still allowing access to deeper brain regions with the native shank geometry.

## 5. Conclusion

Achieving high quality acute and chronic electrophysiological recordings from the brain using penetrating neural probes not only requires innovations in microelectrode array technology but also simultaneous development of more user-friendly surgical methods to reliably implant devices to their intended target. This study addresses the need to advance surgical methods for placing polymer microelectrode arrays having a single shank or multiple shanks in rat. A motorized method to apply a biodegradable coating is presented and compared with previously developed manual dip coating and molded brace methods, including coating process development, coating characterization, mechanical coated device characterization, and *in vivo* demonstration. The findings support that motorized dip coating can assist surgical placement of devices while also being simple to implement for depths up to 5 mm in rat brain.

## Data Availability

The electrophysiological data are available in the DANDI (NIH repository). DANDI: https://dandiarchive.org/dandiset/000675/draft. The data that support the findings of this study are available upon reasonable request from the authors. Part of the data is already available, and the remaining data can be provided upon reasonable request because no suitable repository exists for hosting data in this field of study.
